# Access to Paediatric Essential Medicines: A Survey of Prices, Availability, Affordability and Price Components in Shaanxi Province, China

**DOI:** 10.1371/journal.pone.0090365

**Published:** 2014-03-03

**Authors:** Xiao Wang, Yu Fang, Shimin Yang, Minghuan Jiang, Kangkang Yan, Lina Wu, Bing Lv, Qian Shen

**Affiliations:** 1 Department of Pharmacy Administration, School of Pharmacy, Health Science Center, Xi'an Jiaotong University, Xi'an, China; 2 Department of Population Medicine, Harvard Medical School and Harvard Pilgrim Health Care Institute, Boston, Massachusetts, United States of America; Nottingham University, United Kingdom

## Abstract

**Objective:**

To evaluate the prices and availability of paediatric essential medicines in Shaanxi Province, China.

**Methods:**

Price and availability data for 28 paediatric essential medicines were collected from 60 public hospitals and 60 retail pharmacies in six areas of Shaanxi Province using a standardised methodology developed by the World Health Organization and Health Action International, during November to December 2012. Affordability was measured as the number of days’ wages required for the lowest-paid unskilled government worker to purchase standard treatments for common conditions. Data on medicine price components were collected from hospitals, wholesalers and distributors to obtain price mark-ups.

**Findings:**

The mean availabilities of originator brands (OBs) and lowest-priced generics (LPGs) were 10.8% and 27.3% in the public hospitals and 11.9% and 20.6% in the private pharmacies. The public procurement and retail prices were 2.25 and 2.59 times the international reference prices (IRPs) for three OBs, and 0.52 and 0.93 times for 20 LPGs. In the private sector, the final prices for OBs and LPGs were 3.89 and 1.25 times their IRPs. The final price in the private sector was 2.7% lower than in the public sector for OBs, and 14.1% higher for LPGs. Generally, standard treatments cost less than 1 day’s wages in both sectors. Distribution mark-ups applied to brand salbutamol in Xi'an was 65.5%, and up to 185.3% for generic. Cumulative mark-ups for LPGs in Ankang were also high, from 33% to 50%. The manufacturer’s selling price is the largest contributor to the final price in both areas.

**Conclusions:**

The government should approve a list of national paediatric essential medicines. The availability, price and affordability of these should be improved in both public hospitals and private pharmacies to enable children to obtain effective treatment. Measures should be taken to improve the efficiency of the centralised medicine purchasing system.

## Introduction

According to the World Health Organization (WHO), 6.6 million children under the age of five years died in 2012. The leading causes of death were pneumonia, preterm birth complications, birth asphyxia, diarrhoea and malaria [1]. Almost 20% of deaths in children aged under five years are considered preventable by appropriate use of medicines [2].

Essential medicines are those that satisfy the priority healthcare needs of the population. They should be available within the context of functioning health systems at all times, in adequate amounts and in the appropriate dosage forms [3], [4]. However, very few medicines exist in formulations developed specifically for children, and lack of medicines for children has been a global problem. To increase access to essential medicines for children, the WHO launched the “Better Medicine for Children” initiative and “Make Medicines Child Size” campaign to raise awareness and promote global action [5]. Three editions of the WHO Model List of Essential Medicines for Children (EMLc) have already been published between 2007 and 2011, which are intended for use by children up to 12 years of age [6], [7].

As in many other developing countries, few options exist in China in terms of paediatric drugs and dosage forms, and the list of essential medicines for children has yet to be introduced [8]. The 2009 National Essential Medicine List (NEML), which includes 307 Western and Chinese medications, did not sufficiently meet patients’ basic medical needs, especially for children [9]. Although the newly released 2012 NEML [10] included approximately 200 drugs that could be used for children (including 70 formulations and specifications indicated for paediatric use), the shortage of paediatric medicines is still a serious issue.

Gathering evidence on medicine prices and availability is the first step in improving access to affordable treatment. In May 2003, WHO collaborated with Health Action International (HAI) to develop a standardised method for surveying medicine prices, availability, affordability and price components in selected sectors in low- and middle-income countries [11]. Since then, access to essential medicines has been studied widely using the WHO/HAI methodology [12], but only a small number of studies [13]–[18] in developing countries have been conducted to measure paediatric medicine prices and availability and they did not cover China.

Given the paucity of data available on the availability and cost to the patient of paediatric medicines in China, we conducted a survey in the public and private sectors in six areas of Shaanxi Province, Western China using the WHO/HAI methodology [19] from November to December 2012, to measure the prices and availability of 28 paediatric essential medicines. We also collected price components data for five paediatric essential medicines throughout the supply chain to obtain price mark-ups. To our knowledge, this is the first such paediatric essential medicines survey reported in China.

## Methods

### Ethics statement

The study was approved by the Ethics Committee of Xi'an Jiaotong University Health Science Center. Shaanxi Provincial Department of Health, and Shaanxi Food and Drug Administration approved the study before data collection. The participants were informed of the aims of our study prior to participation. All participants provided signed written informed consent forms.

### Survey areas

For the price and availability survey, Xi’an, the capital city of Shaanxi Province, was chosen as the major urban center, and an additional five areas (Yulin, Baoji, Xianyang, Weinan, and Ankang) within 1 day’s travel of Xi’an were randomly selected.

We investigated price components in two areas: Xi’an (the main urban center) and Ankang (far from the urban center) to collect the price mark-ups in the supply chain.

### Selection of medicine outlets

In each survey area, we first selected the main public hospital, which could be a tertiary hospital. An additional four public sector medicine outlets, two secondary hospitals and two primary hospitals were randomly chosen within 3 h travel of the main hospital. The public sector sample therefore comprised five public sector medicine outlets in each of the six survey areas, a total of 30. The private sector sample also consisted of a total of 30 retail pharmacies, one large-scale pharmacy, two medium-scale pharmacies and two small-scale pharmacies in each area. The selected pharmacies were those closest to each public medicine outlet. In addition to the public and private sector outlets, back-up medicine outlets nearest to, and representing the same level of care as the first ones, were identified in case less than 50% of the medicines were available at the first outlet.

We also recorded information on charges made for five medicines starting at the dispensing point for public hospitals, and tracking price components backwards along the supply chain at different stages. The samples included manufacturers, public hospitals, wholesalers and distributors.

### Selection of survey medicines

A total of 28 medicines were surveyed, all of which were registered in the country. Twenty-one of them were on the WHO’s EMLc [20]. The other seven were selected based on the local disease burden and needs (determined by a pilot survey), the 2009 NEML, Shaanxi Provincial Essential Medicine Supplementary List and the opinions of several groups of experts. Table 1 lists all the survey medicines.

**Table 1:** Availability of medicines in the public sectors and the private sectors.

	Survey medicines	Public sector availability (%)		Private sector availability (%)	
		OBs	LPGs	OBs	LPGs
Core list	Amoxicillin (250 mg, capsule/tablet)	—	58.3	—	78.3
	Amoxicillin/ Clavulanic acid (125 mg+31.25 mg/5 ml, suspension)	—	0	—	0
	Azithromycin (250 mg, tablet)	3.3	33.3	1.7	60.0
	Benzylpenicillin (1 million IU, injection)	—	75.0	—	15.0
	Calamine (100 ml, lotion)	—	70.0	—	71.7
	Carbamazepine (200 mg, tablet)	1.7	0	1.7	3.3
	Cefazolin (1 g, injection)	—	16.7	—	6.7
	Ceftriaxone (250 mg, injection)	3.3	1.7	0	5.0
	Chloramphenicol (250 mg, tablet)	—	8.3	—	13.3
	Chlorpheniramine (4 mg, tablet)	—	68.3	—	75.0
	Diazepam (5 mg/ml, injection)	—	63.3	—	0
	Fluconazole (50 mg,capsule)	5.0	16.7	1.7	30.0
	Ibuprofen (200 mg, tablet)	—	0	—	10.0
	Isoniazid(100 mg, tablet)	—	38.3	—	46.7
	Morphine (10 mg, tablet)	—	8.3	—	0
	Oral rehydration solution (500 ml, oral solution)	—	35.0	—	26.7
	Paracetamol (500 mg, tablet)	0	5.0	3.3	13.3
	Phenobarbital (30 mg, tablet)	—	43.3	—	0
	Phenytoin (50 mg, tablet)	—	26.7	—	15.0
	Procaine penicillin (600 mg, injection)	—	0	—	0
	Salbutamol (100 mcg/dose,inhaler)	31.7	16.7	13.3	33.3
Supplementary list	Albendazole (200 mg, tablet)	31.7	6.7	70.0	6.7
	Aminophylline (25 mg/ml, injection)	—	21.7	—	6.7
	Amoxicillin/ Clavulanic acid (250 mg+125 mg, tablet)	—	6.7	—	10.0
	Beclomethasone (50 mcg/ dose, inhaler)	10.0	0	3.3	0
	Phenobarbital (100 mg/ml, injection)	—	53.3	—	1.7
	Vitamin A (25,000 IU, capsule)	—	1.7	—	8.3
	Vitamin B6 (50 mg/ml, injection)	—	90.0	—	20.0

OBs: Originator brands; LPGs: Lowest-priced generics;

—: Not included in analysis.

For each medicine, price and availability data were collected for two products: the originator brand (OB) and the lowest price generic (LPG) equivalent. The “originator brand” product is a single-brand product marketed by the originator pharmaceutical company. “Lowest price generic equivalents” are defined as the same product sold under the generic name with the lowest unit price at each medicine outlet at the time of data collection in the survey. Twenty medicines were so old that it was not possible to identify the originator brands, so data were only collected for the generics.

For the price components survey, five of the 28 medicines selected had high prices, and/or high use volumes and/or variable pricing patterns. They covered four categories of drug formulations (Table S1). Three of the five were included in the Shaanxi Provincial Procurement List [21] and were purchased through open tender and unified distribution channels.

### Data Collection and Entry

Twelve well-trained data collectors visited medicine outlets and collected data on medicine prices (procurement price and patient price in public sector outlets, and patient price in private sector outlets) and availability, using standardised price collection forms. No specific permits were required for the described field studies.

Two trained personnel entered the data into the standardised WHO/HAI Excel Workbook using a double-entry technique.

Four data collectors visited key informants along the drug supply chain and collected price components data. Then, we entered this information into the WHO/HAI Excel workbook.

### Statistical Analysis

The availability of the investigated medicines was calculated as the percentage (%) of medicine outlets in which the individual medicine was found on the day of data collection. Mean availability refers to the overall ‘‘basket’’ of medicines surveyed.

To facilitate comparisons with other countries, we used the median price ratio (MPR) to express how much greater or less the median local medicine price was than the international reference price (IRP) in the Management Sciences for Health (MSH) 2011 Price Indicator Guide [22], e.g. an MPR of 2, would mean that the local medicine price is twice the international reference price. MSH international reference prices, which are generally offered by not-for-profit suppliers to developing countries, are recommended as the most useful standard. Generally, an MPR of one or less indicates an efficient public sector procurement system and an MPR of 1.5 and two is the cut-off point for patient price in the public and private sector, respectively. Price ratios were not calculated when the medicine was present in fewer than four outlets.

Affordability was estimated to determine the number of days’ wages required to purchase the standardised treatment, using the daily wage of the lowest-paid unskilled government worker. In Shaanxi Province, this was RMB 29.5833 (USD 4.7061) [23]. If a medicine treatment cost less than 1 day’s wages, we regarded its affordability as good.

Data collected on the components of medicine prices were analysed according to key stages in the supply chain. Prices were collected at the following stages: manufacturer’s selling price (MSP), wholesale procurement price, wholesale selling price, public sector procurement price, final patient price and government limited price. We calculated the mark-ups at every stage, and the contribution of each stage to the final price.

## Results

### Medicine availability

The mean availabilities of OBs and LPGs were 10.8% and 27.3% in public hospitals and 11.9% and 20.6% in private pharmacies. The availability of the selected medicines in both sectors was low. For the 21 medicines listed on the EMLc, the mean availability in the public hospitals was 7.5% for OBs and 27.9% for LPGs, and 3.6% for OBs and 25.2% for LPGs in private pharmacies.

Table 1 shows the availability of individual medicines in both the public and private sectors. Seven OBs were found in the public sector, and seven in the private sector. For two medicines, procaine penicillin and amoxicillin/clavulanic acid suspension, neither their originator brands nor generic equivalents were found in any of the survey outlets. Only seven LPGs in the public sector and four in the private sector had >50% availability.

### Medicine prices

Table 2 shows the median MPR of OBs and LPGs in the public and private sectors. In the public sector, the procurement and retail prices were 2.25 and 2.59 times the IRPs for three OBs, and 0.52 and 0.93 times the IRPs for 20 LPGs. The final patient prices were 15.1% and 78.8% higher than procurement prices for three OBs and 20 generic equivalents. The MPRs of three OBs—albendazole (6.24), beclomethasone (2.59) salbutamol (1.75)—were more than 1.5 times their IRPs. Six LPGs were also sold at more than 1.5 times the reference price: fluconazole (5.31), oral rehydration salts (2.22), amoxicillin/clavulanic acid (tablet) (2.10), amoxicillin (1.82), albendazole (1.68) and salbutamol (1.34).

**Table 2:** Median MPRs for OBs and LPGs in the public and private sectors.

Product type	Public sector				Private sector	
	No. of medicines	Median procurement price (MPR)	Median retail price (MPR)	% difference retail to procurement price	No. of medicines	Median retail price (MPR)
LPGs						
All	20	0.52	0.93	78.8	19	1.25
EMLc	15	0.51	0.80	56.9	14	1.20
OBs	3	2.25	2.59	15.1	2	3.89

OBs: Originator brands; LPGs: Lowest-priced generics; MPR: Median price ratio.

EMLc: WHO Model List of Essential Medicines for Children.

In the private sector, the final patient prices for OBs and LPGs were 3.89 and 1.25 times their IRPs. The MPRs of the following medicines were more than twice the IRPs: albendazole (6.00) for OB, and paracetamol (8.79), fluconazole (4.76), albendazole (4.03), oral rehydration salts (3.23) and aminophylline (2.45) for LPGs.

For those medicines found in both public and private sectors, the median MPR for OBs and LPGs were 4.00 and 1.10 in the public sector and 3.89 and 1.26 in the private sector. The final patient price in the private sector was 2.8% lower than in the public sector for OBs, and 14.5% higher for LPGs.

### Affordability

The affordability of standard treatments for eight different health conditions (16 medicines) is calculated. It includes three OBs and 14 LPGs from public sector outlets and two OBs and 12 LPGs from private sector ones. The results showed that standard treatments cost less than 1 day’s wages for both OBs (except for beclomethasone, which cost 1.6 days’ wages) and LPGs in both sectors. As a whole, the affordability was reasonable.

### Price components

In China, no dispensing fee is applied in public hospitals. The transport costs from manufacturers to wholesalers are included in the procurement prices paid by wholesalers. The tax is generally 4%.

Tables 3 and 4 list the price components of five medicines in Xi’an and Ankang. Final patient prices were lower than their government-decided or guided prices. In Xi'an, the total mark-up (from MSP to the final patient price) for salbutamol was 65.5% and 185.3% for OB and generic. The total mark-up on amoxicillin was 77.4%. The mark-ups for generic aminophylline, amoxicillin and oral rehydration salts in public sector outlets were in the range of 20–30%, with retail mark-ups all being around 15%. For two other generic medicines, albendazole and salbutamol, the retail mark-ups were zero (Table 3). In Ankang, for four generic medicines, the cumulative mark-ups were in the range of 30–50%. The retail mark-ups were around 15% for two generics (amoxicillin and oral rehydration salts) and two originator brands (albendazole and salbutamol) and zero for two other generics (albendazole and aminophylline) (Table 4).

**Table 3:** Price components for paediatric medicines in Xi’an.

Medicine	MSP (Yuan)	Wholesale procurement price (Yuan)	Wholesale selling price (Yuan)	Public sector procurement price (Yuan)	Final patient price (Yuan)	% Cumulative mark-up	Government-decided price or government-guided price (Yuan)
LPGs							
Albendazole 200 mg Tablet	2.30	2.30	2.70	2.80	2.80	21.7	6.40
Aminophylline 25 mg/ml Injection	0.54	0.54	0.60	0.60	0.69	27.8	0.84
Amoxicillin 250 mg Capsule	6.20	6.20	6.80	9.60	11.00	77.4	11.50
Oral rehydration 500 ml Oral solution	0.35	0.35	0.36	0.38	0.44	25.7	7.90
Salbutamol 1000 mcg/dose inhaler	12.90	12.90	13.65	36.80	36.80	185.3	52.90
OBs							
Salbutamol 1000 mcg/dose inhaler	14.20	14.20	14.50	20.39	23.50	65.5	23.80

OBs: Originator brands; LPGs: Lowest-priced generics; MSP: manufacturer’s selling price; Yuan: Chinese Yuan.

**Table 4:** Price components for paediatric medicines in Ankang.

Medicine	MSP (Yuan)	Wholesale procurement price (Yuan)	Wholesale selling price (Yuan)	Public sector procurement price (Yuan)	Final patient price (Yuan)	% Cumulative mark-up	Government-decided price or government-decided price (Yuan)
LPGs							
Albendazole 200 mg Tablet	1.40	1.40	2.10	2.10	2.10	50.0	2.50
Aminophylline 25 mg/ml Injection	0.40	0.40	0.53	0.53	0.53	32.5	0.84
Amoxicillin 250 mg Capsule	7.80	7.80	8.80	9.70	11.20	43.6	11.50
Oral rehydration 500 ml Oral solution	32.69	32.69	35.80	37.80	43.50	33.1	44.00
OBs							
Albendazole 200 mg Tablet	9.07	9.07	8.98	9.00	10.40	14.7	11.00
Salbutamol 1000 mcg/dose inhaler	19.2	19.2	20.39	20.39	23.40	21.9	23.80

OBs: Originator brands; LPGs: Lowest-priced generics; MSP: manufacturer’s selling price; Yuan: Chinese Yuan.

For five types of dosage forms in the survey, the cumulative mark-ups varied, with the inhaler having the highest cumulative mark-up in Xi'an and tablet form in Ankang. In the whole supply chain, the MSP was the largest contributor (65.3%) to final patient price in Xi’an, followed by the mark-up from wholesaler to hospital (25.9%), and retail mark-up by hospitals (8.8%). Similarly in Ankang, the relative contributions to the final patient price were the MSP (76.0%), mark-up by wholesalers (15.2%), and mark-up by hospitals (8.8%).

## Discussion

No previous survey has been carried out to assess prices and availability of paediatric essential medicines in China, although some pricing and availability surveys [24]–[28] have been conducted for adult medicines using the validated methodology [19]. This preliminary survey provides a valuable picture of the prices, availability and affordability of essential medicines for children in a reliable and standardised way in China.

The findings reported in this paper focus on the prices and availability of 28 paediatric essential medicines in public hospitals and retail pharmacies of six areas in Shaanxi Province. The results showed that the availability of medicines was low in both sectors. Public sector procurement and patient prices were high for OBs, and both OBs and generics were expensive in private retail pharmacies when compared with the IRPs.

In China, there is still no list of essential medicines for children, and lack of access to paediatric essential medicine has caused growing concern. Strengths and dosage forms suitable for children were in short supply in the market. It is estimated that children account for 20% of the total population in China, but paediatric medicines accounted for only 2% of the total medicines available [29]. In routine clinical treatment, doctors have become used to reducing doses of adult medicines to deal with paediatric diseases. The survey results, however, revealed that the situation might be much more serious than previously thought. The availability of existing OBs and their generic alternatives was lower than 30% in both the public and private sectors, which could be attributed to multiple factors: manufacturers’ lack of motivation to produce because of low demand or small profit, less effective bidding and procurement systems for paediatric medicines, hospitals' preferences for higher-priced medicines, inappropriate drug selection and prescription, and weak health and supply systems [30], [31].

It was noted that no versions of amoxicillin/clavulanic acid suspension and procaine penicillin were found in any of the survey outlets. For the former, the originator brand was not stocked because the manufacturer has removed all domestic amoxicillin/clavulanic acid from the market because of contamination by diisodecyl phthalate [32]. Neither the originator brand nor the generic equivalent of the latter was procured in Shaanxi Province [33]. However, when medicines are not available in hospitals or retail pharmacies, patients will be given substitute medicines, which may not be suitable for them.

By the end of 2011, the Shaanxi provincial government had implemented “triple unification” (unified procurement, unified price and unified distribution) and zero mark-up policies on medicines in all the primary health facilities [34]. Results showed that the retail prices for LPGs were lower in the public sector than in the private sector, to some extent, indicating that the centralised purchasing system plays an active role. However, the overall prices for OBs were much higher than international reference prices and therefore still need to be adjusted. Compared with OBs, the relatively low retail prices for LPGs are probably due to the fierce market competition in China [35]. The results were consistent with a number of pricing and availability surveys [24]–[27] that have been conducted for adult medicines in China.

In this study, we considered affordability of medicines for eight different paediatric health conditions, mostly focusing on acute diseases. This is consistent with actual treatments for children. These standard treatments cost less than 1 day’s wages, because the treatment duration for acute conditions is shorter than for chronic diseases. These findings are different from those of other studies [36]–[38] into affordability of adult medicines, which showed that acute medicines were unaffordable for many people, particularly in private sector outlets. However, treatment for asthma with beclomethasone (costing 1.6 days’ wages) was unaffordable in the public sector. Beclomethasone is used in the ongoing treatment of a chronic condition, making it even less affordable.

Generally, high mark-ups and profit margins were noted in the public sector, while the final patient prices did not exceed the ceiling prices set by the government. However, we recommend urgently that the government should substantially improve public drug procurement and price management, making the procurement system more efficient and the pricing system more scientific, rational and transparent [39].

The present study has several limitations. First, although alternate strengths of the same medicine existed in the survey outlets, they were not recorded in the pre-determined medicine price data collection form. As a result, the medicine availability estimates in this survey may be biased. Second, availability data only apply to the day of data collection and may not reflect average availability over time. Third, limited price data were obtained, because of the low availability of the medicines surveyed. Only two and three originator brand medicines in private and public sectors, respectively, had the required four prices to enable calculation of the MPRs. Low availability of drugs in both sectors therefore makes the median MPR less robust. Finally, this study analysed price components of paediatric essential medicines in China using the standardised WHO/HAI methodology. However, only five medicines were included in the analysis with limited price data collected along the supply chain, because of confidentiality and sensitivity.

## Conclusions

In both the public and private sectors, generic paediatric medicines had better availability than originator brands, but availability was still very low. The affordability was reasonable, although high mark-ups existed along the supply chain for all the paediatric essential medicines surveyed. We recommend that relevant measures should be taken to enable children to obtain sufficient medicines and effective treatment at affordable prices. The government should adjust the prices of originator brands and lowest-priced generics and improve the efficiency of centralised medicine purchasing systems.

## Supporting Information

Table S1 **Price components survey medicines.**
(DOCX)
