# Supplementary material for: Access to Paediatric Essential Medicines: A Survey of Prices, Availability, Affordability and Price Components in Shaanxi Province, China
Source: PLoS One. 2014 Mar 3;9(3):e90365. doi: 10.1371/journal.pone.0090365 (PMC3940867; doi:10.1371/journal.pone.0090365)
Supplement: Table S1 — Price components survey medicines. (DOCX) [file pone.0090365.s001.docx]

**Table S1. Price components survey medicines.**

| Medicine | Dosage form | Strength | WHO's EMLc | Centralized distribution | Disease |
| --- | --- | --- | --- | --- | --- |
| Amoxicillin | capsule/tablet | 250mg | Yes | No | Infection |
| Oral rehydration | oral solution | 500ml | Yes | No | Diarrhoea |
| Albendazole | tablet | 200mg | No | Yes | Intestinal parasite |
| Aminophylline | injection | 25mg | No | Yes | Asthma |
| Salbutamol | inhaler | 100mcg/dose | Yes | Yes | Asthma |
